# The Effect of Physical Activity on Anterior Segment Structures and the Retinal Nerve Fiber Layer: A Comparison of Elite Athletes and Sedentary Individuals

**DOI:** 10.3390/medicina61091623

**Published:** 2025-09-08

**Authors:** Çiğdem Deniz Genç, Esra Korkmaz Salkılıç, Berna Anıl, Enes Akdemir, Coşkun Yılmaz, Ali Kerim Yılmaz

**Affiliations:** 1 Department of Ophthalmology, Faculty of Medicine, Samsun University, Samsun 55090, Türkiye; cigdem.deniz@samsun.edu.tr; 2 Faculty of Yaşar Doğu Sport Sciences, Ondokuz Mayıs University, Samsun 55270, Türkiye; esrkmz@gmail.com (E.K.S.); akdemrenes@gmail.com (E.A.); akerim.yilmaz@omu.edu.tr (A.K.Y.); 3 Kelkit Aydın Doğan Vocational School, Gümüşhane University, Gümüşhane 29600, Türkiye; coskun.yilmaz@gumushane.edu.tr

**Keywords:** retinal nerve fiber layer, anterior segment structure, intraocular pressure, elite individual athletes

## Abstract

*Background and Objectives:* The study aims to evaluate the effects of regular physical activity on ocular structures by comparing eye anterior segment structures and retinal nerve fiber layer (RNFL) parameters between elite individual athletes and sedentary persons. *Materials and Methods:* This cross-sectional observational study included 75 participants (33 female and 42 male) aged 18–32 years. Participants consisted of 33 elite individual athletes (66 eyes) aged 18–32 years and 42 sedentary individuals (84 eyes) aged 18–28 years. The elite athlete group consisted of participants who had trained ≥ 3 times per week for the past 5 years and had competition experience in tennis (n = 11), badminton (n = 8), and short/middle distance running (n = 14). Sedentary participants had not engaged in regular physical activity for the last 2 years. Anterior segment structures were measured with ultrasound biomicroscopy (UBM), RNFL parameters were measured with spectral-domain optical coherence tomography (SD-OCT), and intraocular pressure (IOP) was measured with a non-contact pneumotonometer. *Results:* When the anterior segment parameters were compared, a significance was found in the group effect (*p* = 0.021, ${ƞ}_{p}^{2}\;$ = 0.036) in the TIA500 value, but the effects of side and gender were insignificant (*p* > 0.05). While the gender effect was found to be significant for the AOD500 (*p* = 0.030, ${ƞ}_{p}^{2}\;$ = 0.032) and ARA500 (*p* = 0.019, ${ƞ}_{p}^{2}\;$ = 0.038) variables, the group and side effects were statistically insignificant (*p* > 0.05). There was a significant difference in IOP values between sedentary individuals (15.82 ± 2.69) and athletes (14.61 ± 1.80) (*p* = 0.004, ${ƞ}_{p}^{2}\;$ = 0.057). There was a significant difference between the right (15.71 ± 2.70) and left (14.87 ± 2.02) sides (*p* = 0.027, ${ƞ}_{p}^{2}\;$ = 0.033). The effect of gender was not significant (*p* > 0.05). When the results were evaluated, a significant effect of the TEMPORAL side in RNFL parameters (*p* = 0.003, ${ƞ}_{p}^{2}$ = 0.058) was observed. Correlations were seen between the anterior segment and RNFL parameters in both groups (*p* < 0.05). *Conclusions:* Athletes exhibited lower IOP compared with sedentary individuals, with similar RNFL parameters but distinct correlation patterns with anterior segment structures. These findings suggest that ocular parameters should be evaluated in an integrated manner and that physical activity may support ocular health by contributing to IOP reduction and potentially slowing the progression of eye diseases.

## 1. Introduction

In addition to the positive effects of physical activity on neurodegenerative and cardiovascular diseases, its effects on ocular health parameters such as glaucoma, axial length, ocular blood flow, and myopia, as well as systemic diseases such as type 2 diabetes mellitus and hypertension, have also been investigated [1,2]. While the benefits of regular physical activity on general health have been extensively studied, its effects on eye structure and function are drawing more interest.

In athletes, physical activity requiring high levels of visual attention, peripheral awareness, and rapid reaction may lead to various adaptations in the visual system, resulting in changes in ocular biometric parameters [3,4]. The positive effects of exercise on eye health are increasingly recognized and confirmed by a growing number of studies [5,6,7,8]. In eye health, mechanisms such as reducing chronic oxidative stress, reducing inflammation, improving mitochondrial function, increasing ocular blood circulation, and releasing protective factors are highlighted in conjunction with daily and regular exercise [9]. These adaptations may create differences in RNFL thickness and anterior segment parameters [10,11,12]. In the literature, significant differences in many parameters such as intraocular pressure (IOP), axial length, central corneal thickness, anterior chamber depth, and retinal nerve fiber layer (RNFL) thickness have been reported in individuals with different physical activity levels [1,2,13,14]. In addition, dynamic visual acuity has been reported to be higher in athletes compared with sedentary persons [15]. One of the most frequently reported exercise-related effects is a decrease in IOP levels with the duration and intensity of exercise [16,17,18,19,20]. Possible mechanisms of these changes include decreased aqueous humor production due to increased plasma osmotic pressure because of sweating and fluid loss during exercise, choroidal vasoconstriction due to increased peripheral circulation redistributing intraocular blood flow, and sympathetic nervous system activation [17,21,22,23,24]. It has also been suggested that increased catecholamine levels due to exercise may increase aqueous humor outflow from the trabecular meshwork.

Spectral-domain optical coherence tomography (SD-OCT) has been widely used to evaluate changes in these parameters, especially in the analysis of RNFL thickness. SD-OCT is a non-invasive imaging modality that can cross-sectionally visualize retinal tissues and the optic nerve head at high resolution [25,26]. On the other hand, ultrasonic biomicroscopy (UBM), one of the leading methods for evaluating anterior segment structures, is a technique that requires direct contact with the eye and uses ultrasound in the 50–100 MHz frequency range [27]. It can produce high-resolution cross-sectional images of the anterior segment in living tissue and provide objective quantitative measurements of anterior segment parameters [28] and is advantageous because it allows quantitative measurements to be obtained [27,29]. The high-resolution results obtained with SD-OCT and UBM, and especially the quantitative and objective assessment of the anterior segment in living tissue with UBM, suggest that clearer and more reliable results for ocular biometric differences can be obtained by using both methods together.

This study aims to evaluate the effects of regular physical activity on ocular structures by comparing anterior segment structures and RNFL parameters between athletes and sedentary individuals of a similar age group. A more comprehensive study on the effects of physical activity on ocular biometry may contribute to the customization of ophthalmologic health screenings according to individual characteristics and may pave the way for the development of approaches to protect eye health in athletes. Considering the contradictory findings in the literature, this study was conducted to investigate whether athletes who engage in regular physical activity differ from sedentary individuals in terms of anterior segment parameters, IOP levels, and RNFL thickness.

## 2. Materials and Methods

### 2.1. Participants

This study included a total of 75 participants, 33 female and 42 male, aged 18–32 years. The participant group consisted of 33 elite individual athletes (66 eyes) and 42 sedentary individuals (84 eyes). The elite athlete group included 11 tennis players (6 female, 5 male), 8 badminton players (4 female, 4 male), and 14 short- and medium-distance runners (8 female, 6 male).

Recruitment was conducted prospectively at Samsun Training and Research Hospital between January 2024 and January 2025. The optimal number of subjects was determined using the G*Power 3.1.3 program. To determine the sample size, the minimum number of participants required for covariance analysis with two groups, two eye directions, and two genders, adjusted for age, was calculated using effect sizes. Based on a priori power analysis (ANCOVA), the minimum required sample size was 68 participants (f = 0.40, α = 0.05, and power (1 − β) = 0.90).

All athletes had a minimum of 5 years of continuous training and competition, a criterion selected to ensure sufficient chronic physiological adaptation that could influence ocular structures, particularly the RNFL. Athletes trained ≥ 3 times per week, at least 100 min per session, at moderate to high intensity, and had actively competed at club or national levels within the last 1 year. The sedentary group included age-matched individuals (18–32 years) who had not engaged in any regular physical activity in the last 2 years and had no ocular or systemic conditions that could affect measurements.

Informed consent was obtained from all participants after providing a detailed explanation of the study procedures.

### 2.2. Study Design

This cross-sectional observational study was approved by the local institutional ethics committee (Approval number: E-95674917-108.99-239701) and conducted in accordance with the principles outlined in the Declaration of Helsinki.

Inclusion criteria for the athlete group were being an elite individual athlete aged 18–32 years with at least 5 years of sports history, no history of ocular trauma or surgery, and absence of ocular pathology that could affect OCT and UBM imaging. Inclusion criteria for the sedentary group were age 18–32 years, no ocular pathology, no trauma or surgery, and refractive error within ±1.0 diopter.

Exclusion criteria for both groups included age < 18 years, previous ocular surgery or trauma, refractive error > ±1.0 diopter, axial length > 26 mm, presence of diseases such as glaucoma or uveitis, or amblyopia in any eye.

To ensure measurement reliability and minimize observer-related variability, all assessments were performed by a single experienced ophthalmologist. Measurements were taken for all participants during the same time window in the afternoon (14:00–16:00) to control for potential diurnal fluctuations. Participants were instructed to avoid heavy meals and caffeinated beverages for at least 12 h prior to measurements, maintain normal hydration, and refrain from intense physical activity in the 24 h before testing.

### 2.3. Procedures

A comprehensive systemic and ophthalmologic history was recorded for all participants. All patients underwent a detailed ophthalmologic examination, including best corrected visual acuity (BCVA) with Snellen chart, intraocular pressure (IOP) and central corneal thickness (CCT) measurements with a non-contact pneumotonometer (Topcon CT-1P), biomicroscopic anterior segment and dilated fundus examinations, and strabismus examination with Hirshberg and cover/uncover tests. Ocular motility was assessed in nine cardinal directions of gaze. Refractive error and keratometry values were measured with an autorefractometer (Topcon KR-8100P, Tokyo, Japan). Refractive errors were converted to spherical equivalent (SE) values for analysis. SE ≤ −1 D was defined as myopia, SE between −1 and +1 D as emmetropia, and SE ≥ +1 D as hyperopia. Anisometropia was defined as an SE difference of ≥ 1 D between the two eyes of the patient. Astigmatism was defined as a cylindrical refractive error of ≥ 1.0 diopter.

**Figure 1:**
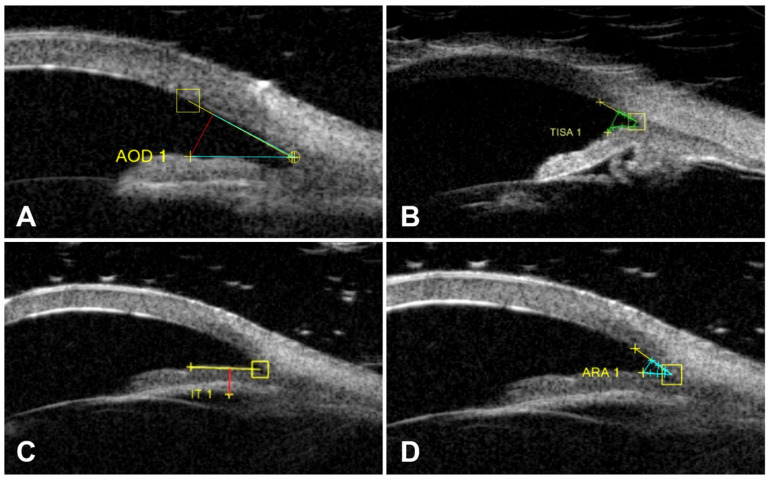
Biometric measurements of ocular structures: (**A**) angle opening distance, (**B**) trabecular–iris surface area, (**C**) iris thickness, (**D**) angle recess area. 1. Angle opening distance 500 (AOD500) is the distance between the inner corneal surface and the anterior iris surface measured on a line perpendicular to the plane of the trabecular meshwork at 500 μm from the scleral spur. 2. The trabecular–iris angle (TIA) was measured with the apex of the angle at the iris recess and the arms of the angle passing through a point on the trabecular meshwork 500 μm from the scleral spur and a point perpendicular to the iris. 3. Trabecular–iris surface area at 500 μm and 750 μm and TISA 750: We propose this new parameter for quantitative measurement of the AC angle. The defining boundaries for this trapezoidal area are as follows: AOD 500 or AOD 750 at anterior; a line drawn from the scleral spur to the opposite iris perpendicular to the plane of the inner scleral wall at posterior; the inner corneoscleral wall at superior; and the iris surface at inferior. TISA excludes the non-filtering area behind the scleral spur. 4. Iris thickness (IT) was measured at 500 μm from the iris root.

UBM 10 mHz and 50 mHz ultrasound probes (Aviso Quantel Medical, Clermont-Ferrand, France) were used for biometric measurements of eye structures. UBM examinations were performed with the patient in a sitting position, under constant room lighting, and by asking them to look at a fixed point from a distance of 3 m (to keep accommodation constant). Topical proparacaine HCl 0.5% (Alcaine^®^, Alcon, Zug, Switzerland) was administered for topical anesthesia before measurements. The UBM 50 mHz probe was placed in contact with the eye with the eyelids open. Axial images of the anterior segment were taken. To ensure consistency and optimal image quality, care was taken with image alignment and vertical orientation during axial scanning of the anterior segment. To evaluate the iridotrabecular angle more clearly, care was taken to select the images with the best reflection of the iris. For accurate and easy localization of the scleral spur, it was ensured that the reflex of the interface between the ciliary body and the sclera was prominent, and the appearance of the ciliary body and iris was good. Trabecular–iris angle (TIA) 500, angle opening distance (AOD) 500, trabecular–iris surface area (TISA) 500, angle recess area (ARA) 500, and iris thickness (IT) 750 were calculated semi-automatically from temporal angle images using scales in the device software (Figure 1).

Spectral-domain optical coherence tomography (SD-OCT) (Topcon Inc., Tokyo, Japan) was employed in this study. Retinal nerve fiber layer (RNFL) thickness was obtained from all 4 peripapillary quadrants (temporal, superior, nasal, and inferior) as well as from 12 sub-quadrants. Circular OCT tomograms were acquired around the optic disc with a diameter of 3.4 mm. The RNFL thickness was displayed on a circular chart divided into four equal 90° sectors, each representing one quadrant and showing thickness values (in micrometers) within the respective sector.

The SD-OCT system operates at a central wavelength of 850 nm with an image acquisition rate of 50,000 A-scans per second. The RNFL boundaries were automatically segmented, and thickness was calculated throughout the scan. A thickness map was generated within a 12 × 9 mm field using color scales corresponding to numeric RNFL measurements. Abnormal findings were assessed by comparing both RNFL and macular [GC-IPL + RNFL] thickness values with the built-in normative database, and deviations were visualized on the SuperPixel deviation map. This map consists of a peripapillary RNFL deviation plot with 26 × 26 pixels within a 5.2 × 5.2 mm^2^ field, where each pixel side measures 200 μm. Uncolored pixels represent values within the normal range, while yellow and red pixels indicate abnormalities at the < 5% and < 1% levels, respectively.

A wide-field RNFL defect was defined as an arcuate or wedge-shaped, diverging dark-blue region characterized by an abrupt change in the color scale, indicating reduced thickness compared with adjacent areas. The minimum defect size considered was larger than the diameter of a major retinal vessel.

Images were taken and measurements were performed by the same physician. Measurements from both eyes of all participants in each group were included in the analysis.

### 2.4. Statistical Analysis

The normality assumption of the data was examined using the Kolmogorov–Smirnov test, and all variables showed normality (*p* > 0.05). The differences in age, AL, and BCVA between groups were evaluated using the independent samples *t*-test. Since a significant difference was observed when the effect of age was taken into account and the data showed a normal distribution, covariance analysis (ANCOVA) was applied to examine the effects of group, side, and gender after adjusting for age. The model used for the dependent variables was as follows:

$$Y_{ijkl}=µ+\alpha_{i}+\gamma_{j}+\theta_{k}+\beta \left(X_{ijkl}\right)+\varepsilon_{ijkl}$$

Here: $Y_{ijkl}:$ observed value; µ: population mean; $\alpha_{i}:$ effect of the group (sedentary/athlete); $\gamma_{j}:$ effect of the *j*^th^ side (right/left); $\theta_{k}$: effect of the *k*^th^ gender (male/female); β: the regression coefficient of age affecting the dependent variable; $X_{ijkl}$: age information; and $\varepsilon_{ijkl}$: random error. The results were presented as mean and standard deviation, and all tests were evaluated at a significance level of *p* < 0.05. In addition, gender distributions were reported as frequencies and percentages. Partial eta-squared values were calculated for the effect values examined in the study. Pearson correlation analysis was performed to determine the direction and strength of the relationship between variables; the magnitude and direction of the correlation coefficient *r* were evaluated for reporting purposes. The categories of the *r* value were as follows: < 0.20 very weak; 0.20–0.39 weak; 0.40–0.59 moderate; 0.60–0.79 strong; ≥ 0.80 very strong relationship. All statistical analyses were performed using IBM SPSS 21.0 (IBM Corp., Armonk, NY, USA) software package, and GraphPad Prism 10.4.1 (GraphPad Software, San Diego, CA, USA) software was used to visualize the correlation table.

## 3. Results

Table 1 summarizes the descriptive characteristics of the athletic and sedentary groups. A significant difference in age was observed in sedentary and athletic groups (*p* = 0.031). Also, in terms of gender, a statistically significant difference was found between the groups in terms of age in men (*p* = 0.023). The results for other parameters were not statistically significant (*p* > 0.05).

The effects of group, party, and gender on the IT750, TISA500, and CCT variables were found to be statistically insignificant (Table 2). The group effect was found to be significant in the TIA500 variable, while the effects of side and gender were insignificant (*p* > 0.05). When the group effects on TIA500 were examined, the value of the sedentary group was higher than that of the athlete group (*p* = 0.021). The effect value of this variable was determined as 0.036 (almost medium level). The group and side effects for the AOD500 (*p* = 0.030) and ARA500 (*p* = 0.019) variables were statistically insignificant. However, the effect of gender was found to be significant in both variables (*p* < 0.05). According to the findings, the values for AOD500 and ARA500 were found to be higher for men than for women. In addition, the effect values for both variables were found to be in the range of 0.032 to 0.038. The group effect (*p* = 0.004) and side effect (*p* = 0.027) were found to be significant in the IOP variable (*p* < 0.05), while the effect of gender was insignificant (*p* > 0.05). While the sedentary IOP value was found to be higher than that of athletes, it was determined that the IOP value on the right side was higher than that obtained on the left side. In addition, while the group’s effect value was almost moderate (0.057), the effect value of the side was low (0.033).

**Figure 2:**
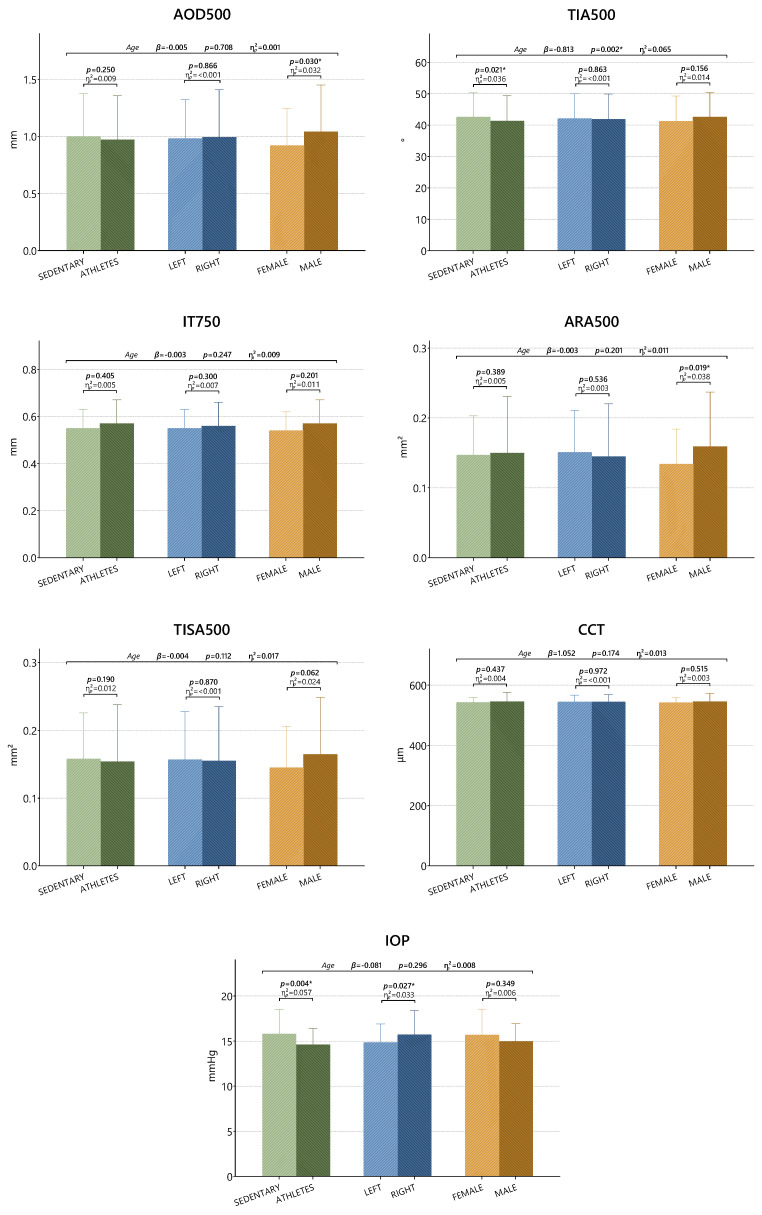
Comparison of anterior segment data in athletes and sedentary subjects. * *p* < 0.05; β: regression coefficient; ${ƞ}_{p}^{2}$: partial eta square effect value; AOD: angle open distance; TIA: trabecular iris angle; IT: iris thickness; ARA: angle recess area; CCT: central corneal thickness; IOP: intraocular pressure.

In Figure 2, gender effects can be observed for the AOD500 parameter (*p* = 0.030), group effects for the TIA500 parameter (*p* = 0.021), gender effects for the ARA500 parameter (*p* = 0.019), and both group (*p* = 0.004) and side effects (*p* = 0.027) for the IOP parameter (*p* < 0.05). For other parameters, group, side, and gender effects were found to be statistically insignificant (*p* > 0.05). Regression analysis revealed that age had a significant and negative effect on TIA500 (β = −0.813) (*p* < 0.05). No significant relationship was found with age for other parameters (*p* > 0.05).

The comparison of anterior segment parameters between the athlete and sedentary groups is presented in Table 3. When the results were evaluated, a significant effect of side (*p* = 0.003, ${ƞ}_{p}^{2}$ = 0.058) was observed in the TEMPORAL parameter (*p* < 0.05). The effects of group, side, and gender were found to be statistically insignificant in the other parameters (*p* > 0.05).

**Figure 3:**
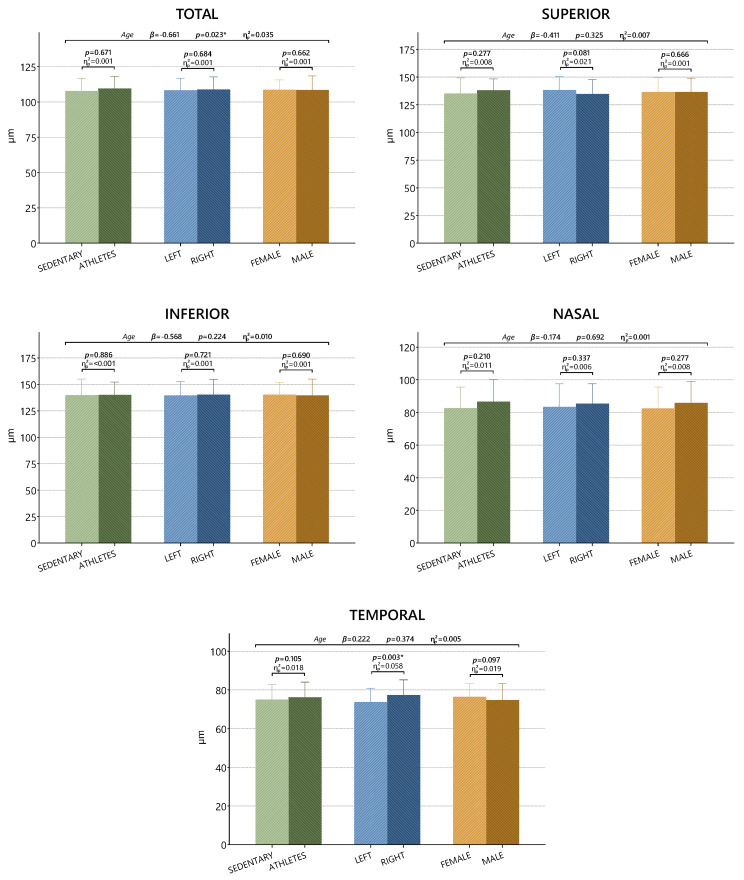
Comparison of RNFL parameters in athletes and sedentary subjects. * *p* < 0.05; β: regression coefficient; ${ƞ}_{p}^{2}$: partial eta square effect value.

A comparison of RNFL values is shown in Figure 3. A side effect was observed in the TEMPORAL parameter (*p* = 0.003) (*p* < 0.05). For other parameters, the effects of group, side, and gender were found to be statistically insignificant (*p* > 0.05). In the regression analysis, age was found to have a significant and negative effect on the TOTAL (β = −0.671) (*p* < 0.05). No significant relationship was found with age for the other parameters (*p* > 0.05).

**Figure 4:**
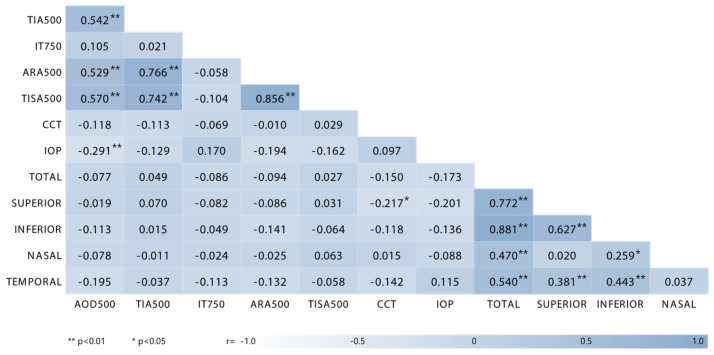
Relationship between anterior segment and RNFL parameters in the sedentary group. AOD: angle open distance; TIA: trabecular–iris angle; IT: iris thickness; ARA: angle recess area; CCT: central corneal thickness; IOP: intraocular pressure. * *p* < 0.05; ** *p* < 0.01; *r*: Pearson correlation.

The relationship between sedentary individuals and anterior segment parameters is examined in Figure 4. When the results were evaluated, it was seen that there was a moderate correlation between AOD500 and TIA500, ARA500 and TISA500 (*r* = 0.542, 0.529, 0.570), a strong correlation between TIA500 and ARA500 and TISA500 (*r* = 0.766, 0.742), a very strong correlation between ARA500 and TISA500 (*r* = 0.856), a strong correlation between TOTAL and SUPERIOR (*r* = 0.772), a very strong correlation between TOTAL and INFERIOR (*r* = 0.881), a moderate correlation between TOTAL and NASAL and TEMPORAL (0.470, 0.540), a strong correlation between SUPERIOR and INFERIOR (*r* = 0.627), a weak correlation between SUPERIOR and TEMPORAL (*r* = 0.381), a weak correlation between INFERIOR and NASAL (*r* = 0.259). A weak positive correlation was observed between INFERIOR and TEMPORAL (*r* = 0.443) (*p* < 0.05), and a weak negative correlation was observed between AOD500 and IOP (*r* = −0.291) and between CCT and SUPERIOR (*r* = −0.217) (*p* > 0.05).

**Figure 5:**
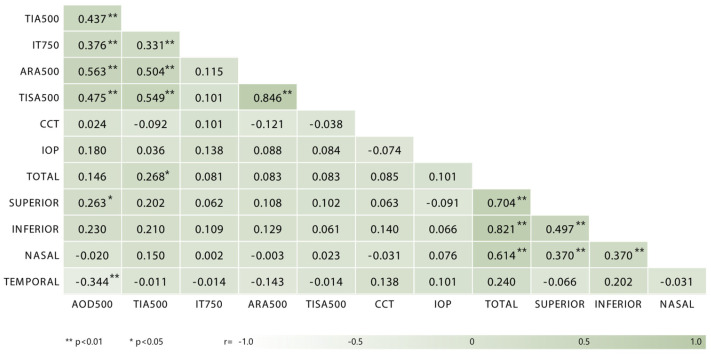
Relationship between anterior segment and RNFL parameters in the athlete group. AOD: angle open distance; TIA: trabecular–iris angle; IT: iris thickness; ARA: angle recess area; CCT: central corneal thickness; IOP: intraocular pressure. * *p* < 0.05; ** *p* < 0.01; *r* Pearson correlation.

The relationship between the athletes and the anterior segment parameters is examined in Figure 5. When the results were evaluated, it was seen that there was a moderate correlation between AOD500 and TIA500, ARA500 and TISA500 (*r* = 0.437, 0.563, 0.475), a weak correlation between AOD 500 and IT750 AND SUPERIOR (*r* = 0.376, 0.263), a weak correlation between TIA500 and IT750 and TOTAL (*r* = 0.331, 0.268), a moderate correlation between TIA500 and ARA500 and TISA500 (*r* = 0.504, 0.549), a very strong correlation between ARA500 and TISA500 (*r* = 0.846), a strong correlation between TOTAL and SUPERIOR and NASAL (*r* = 0.704, 0.614), a very strong correlation between TOTAL and INFERIOR, a moderate correlation between SUPERIOR and INFERIOR (*r* = 0.497). A weak positive correlation was observed between SUPERIOR and NASAL (*r* = 0.370), and a weak negative correlation was observed between INFERIOR and NASAL (*r* = 0.370) (*p* < 0.05). A weak negative correlation was observed between AOD500 and TEMPORAL (*r* = −0.344) (*p* > 0.05).

## 4. Discussion

The main findings of the study are as follows: In the anterior segment data of the athlete and sedentary groups, the IOP value was lower in the athletes. Correlation analysis showed that there were different levels of correlation between anterior segment structures and RNFL parameters in both groups. Although RNFL thickness was similar between the groups, the correlations observed with anterior segment parameters revealed the necessity to evaluate ocular structures in structural integrity.

It is known that regular physical activity provides positive effects on the ocular system as well as general body health [10]. Exercise has also been reported to reduce IOP in the literature [30]. Li et al. (2018) [31] reported a significant decrease in IOP, AOD500, and TISA500 parameters after exercise. Gene-Morales et al. (2022) [32] reported that performing squat exercise with either elastic bands or weight plates was associated with lower IOP, while no change was observed in CCT parameters. In their hypobaric hypoxia-based experimental study, Xie et al. (2025) [33] reported an increase in AOD500 and TISA500 parameters, while structural parameters such as CCT did not change. This increase is a positive indicator of aqueous humor drainage, indicating that the anterior chamber angle has widened and the trabecular meshwork has become more accessible [34,35]. In our study, it was observed that IOP levels were lower in individuals who performed regular sports compared with those who did not, indicating that this may be related to ocular hemodynamic adaptations rather than a causal effect [36]. Although the physiological mechanisms underlying exercise-induced IOP reductions have not been fully elucidated, several possible explanations have been suggested in the literature. These include the possibility that sweating and fluid loss during exercise may increase plasma colloidal osmotic pressure, thereby decreasing aqueous humor production [17], and that blood flow may be diverted primarily to working muscles, leading to transient ischemia in the eye, which may reduce aqueous humor production [21,37,38]. It has also been stated in the literature that IOP may show short-term decreases depending on the intensity of exercise, but regular physical activity can provide a more permanent decrease in basal IOP [39]. However, these mechanisms should still be presented as possible explanations; prospective, controlled, long-term studies are required for definitive proof of causality. When the clinical significance of this statistically significant difference was examined, the difference in IOP between the groups appeared limited when evaluated with small and medium effect sizes, but large-scale prospective studies indicated that every 1 mmHg decrease in IOP reduced the risk of developing glaucoma by approximately 10–14% [40,41]. Therefore, the lower IOP values observed in athletes may provide a protective advantage against glaucoma in the long term [42]. In our current study, to eliminate the acute effects of exercise, athletes refrained from intense exercise for 24 h before measurement. This supports the idea that our cross-sectional study indicates that regular exercise has a positive chronic effect on decreases in IOP rather than its acute effects, and that regular exercise mentioned in the literature can provide a permanent decrease in baseline IOP values. However, due to the cross-sectional design of the study, prospective, long-term follow-up studies are needed to clearly demonstrate the clinical results. Previous studies have reported that exercise-induced changes in iris position and morphology may influence anterior segment parameters [43]. Haargaard et al. (2001) [36] reported an increase in iris concavity and anterior chamber angle width after physical activity, suggesting that exercise may lead to transient but measurable changes in anterior segment structures. Enhanced circulation and reduced IOP following exercise may contribute to improved perfusion of posterior segment structures, including the retina and optic nerve head [44]. In our study, TIA500 values were found to be lower in athletes than in sedentary individuals. This is thought to be due to adaptive changes in ocular fluid dynamics and ciliary muscle–zonule interactions that develop due to regular physical activity [45]. Additionally, the higher AOD500 and ARA500 values in men than in women may be related to the wider anterior chamber angle and larger trabecular iris area in men [46]. One study reported that AOD500 and TISA500 values were significantly higher in men than in women [47]. Similarly, a study with 8-year-old children found that boys had higher anterior chamber parameters than girls [48]. In our current study, it was observed that the other anterior segment parameters are largely similar. This variability may be attributed to differences in exercise duration, intensity, or modality [49]. It is also known that the architecture of the anterior segment is predominantly influenced by genetic factors, age, and inherent ocular morphology rather than transient physiological changes [50,51].

RNFL thickness is known to be influenced by factors such as age, sex, ethnicity, and optic disc morphology, with limited susceptibility to short-term external interventions [52]. Although the difference was higher in athletes in our study’s intergroup evaluation, this difference was not statistically significant. The effect on side and gender was statistically similar. Kawano et al. (2017) [53] stated that vascular density increased in the optic nerve head and macula region, but no significant change was observed in RNFL thickness. However, Mauget-Faÿsse et al. (2021) [11] reported a transient increase in RNFL thickness in their measurements after a marathon and attributed this to exercise-induced hemodynamic changes. It has been suggested that high-intensity exercise may induce temporary morphological alterations in the RNFL due to exercise-related morphological changes [12]. In our study, different patterns were observed in the correlations between RNFL thickness and anterior segment parameters in both the athlete and sedentary groups. A moderate correlation was observed between TIA500 and ARA500 and TISA500, a very strong correlation was observed between ARA500 and TISA500, a strong correlation was observed between TOTAL and SUPERIOR and NASAL, a very strong correlation was observed between TOTAL and INFERIOR RNFL, and a moderate correlation was observed between SUPERIOR and INFERIOR. Moderate correlations seen in athletes may indicate that regular physical activity may affect the structural integrity and ocular hemodynamic adaptations between anterior segment structures and the RNFL [54]. The strong correlation between TOTAL and SUPERIOR and NASAL may reflect the protective effect of regular physical activity on the retinal structure and ocular hemodynamics and may be associated with more balanced perfusion and possible resistance to early glaucomatous changes [55]. In the sedentary group, moderate correlations were observed between AOD500 and TIA500, ARA500 and TISA500, strong correlations between TIA500 and ARA500 and TISA500, very strong correlations between ARA500 and TISA500, strong correlations between TOTAL and SUPERIOR, very strong correlations between TOTAL and INFERIOR, moderate correlations between TOTAL and NASAL and TEMPORAL, and strong correlations between SUPERIOR and INFERIOR. A high correlation between SUPERIOR and INFERIOR indicates that retinal integrity is preserved [56]. For other parameters, correlations were weaker or not significant, indicating that individual biological and anatomical differences play an effective role. Wang et al. (2013) [57], in their study of 1654 individuals, demonstrated that the factors affecting RNFL thickness are individual and anatomical. The very strong correlation between ARA500 and TISA500 in both groups reflects the mutually reinforcing structural integrity of anterior segment parameters and retinal health [34]. The very strong correlation observed between TOTAL and INFERIOR RNFL in both groups indicates that the inferior region exhibits a natural harmony with the total RNFL due to its anatomically dense nerve fiber structure and that this structural condition is preserved regardless of sports status [58]. These findings support the importance of a holistic evaluation approach in holistically assessing eye health; that is, interactions among IOP, anterior segment parameters, and RNFL thickness may have clinical significance in terms of retinal and optic nerve head perfusion and ocular fluid dynamics [59,60]. However, due to the cross-sectional design of the study, causality cannot be inferred, and prospective, long-term studies are necessary. These results support the associations between sports and ocular hemodynamics previously reported in the literature [41,57,61].

This study has some limitations. The study sample consisted only of healthy individuals and elite athletes active in individual sports in the 18–32 age range. This restricts the generalizability of the findings to broader populations. Another limitation is that the measurements were performed in a single period, making it difficult to distinguish between acute and chronic effects of exercise. Although the time interval during which the measurements were taken was the same for all patients, it is thought that the long study period may have affected the results. The inclusion of only healthy participants limits the applicability of the results to clinical populations. The cross-sectional design of our study does not allow us to establish causality in the relationship between regular physical activity and ocular parameters. Findings were evaluated at a correlational level; longitudinal or intervention-based studies are needed to determine the direct effects of exercise. Furthermore, although homogeneity was achieved in the distribution of sports, specific differences between branches may have affected the results. Another limitation is that repeated measurements were not made by the same observer at different times, and intra-observer reliability could not be evaluated. Considering these limitations, further studies can be conducted with larger, heterogeneous samples by including different age and branch groups. At the same time, studies comparing both acute and chronic interventions are needed to distinguish between the acute and chronic effects of exercise.

## 5. Conclusions

The study results demonstrated that athletes had lower IOP compared with sedentary individuals. While RNFL parameters were similar between the groups, the observed correlations between RNFL and anterior segment structures—particularly in athletes—highlight the importance of evaluating these ocular parameters collectively rather than in isolation. This integrated perspective may reveal associations that are not apparent when each parameter is considered separately. The findings also suggest that future longitudinal or interventional studies could clarify how different acute or chronic exercise loads influence both anterior segment and RNFL parameters. Furthermore, given that physical activity enhances blood circulation not only systemically but also within the retina and other ocular structures, exercise may contribute to IOP reduction and serve as an important adjunct in the prevention or slowing of progression of ocular diseases such as glaucoma, diabetic retinopathy, macular degeneration, and cataracts.

## Figures and Tables

**Table 1:** Descriptive characteristics of the groups.

		Athletes (n = 33)	Sedentary (n = 42)	
		Mean *±* SD	Mean ± SD	*p*
**Age (year)**	**F**	21.44 ± 1.69	22.75 ± 2.28	0.135
	**M**	21.04 ± 3.14	23.07 ± 1.98	0.023 *
	**Total**	21.15 ± 2.83	22.88 ± 2.17	0.004 *
**Gender (n/%)**	**F**	9 (27.27)	24 (57.14)	
	**M**	24 (72.72)	18 (42.86)	
**AL (mm)**		22.90 ± 1.16	22.86 ± 1.16	0.834
**BCVA (logMAR)**		−0.06 ± 0.09	−0.07 ± 0.08	0.538

* *p* < 0.05; SD: standard deviation; F: female; M: male; AL: axial length; BCVA: best corrected visual acuity.

**Table 2:** Comparison of anterior segment data in athletes and sedentary subjects.

	Sedentary (n = 33)	Athletes (n = 42)	ANCOVA	
Group	Mean ± SD	Mean ± SD	*p*	${ƞ}_{p}^{2}$
**AOD500 (mm)**	0.99 ± 0.37	0.97 ± 0.39	0.250	0.009
**TIA500 (°)**	42.57 ± 7.73	41.28 ± 8.08	0.021 *	0.036
**IT750 (mm)**	0.55 ± 0.08	0.57 ± 0.10	0.405	0.005
**ARA500 (mm^2^)**	0.15 ± 0.06	0.15 ± 0.08	0.389	0.005
**TISA500 (mm^2^)**	0.16 ± 0.07	0.15 ± 0.08	0.190	0.012
**CCT (μm)**	543.93 ± 15.03	546.15 ± 30.25	0.437	0.004
**IOP (mmHg)**	15.82 ± 2.69	14.61 ± 1.80	0.004 *	0.057
	**Right (n= 75)**	**Left (n= 75)**	**ANCOVA**	
**Side**	**Mean ± SD**	**Mean ± SD**	***p***	${ƞ}_{p}^{2}$
**AOD500 (mm)**	0.99 ± 0.42	0.98 ± 0.34	0.866	<0.001
**TIA500 (°)**	41.89 ± 8.00	42.11 ± 7.82	0.863	<0.001
**IT750 (mm)**	0.56 ± 0.10	0.55 ± 0.08	0.300	0.007
**ARA500 (mm^2^)**	0.15 ± 0.08	0.15 ± 0.06	0.536	0.003
**TISA500 (mm^2^)**	0.155 ± 0.08	0.16 ± 0.07	0.870	<0.001
**CCT (μm)**	544.97 ± 23.61	544.84 ± 22.41	0.972	<0.001
**IOP (mmHg)**	15.71 ± 2.70	14.87 ± 2.02	0.027 *	0.033
	**Female (n = 33)**	**Male (n = 42)**	**ANCOVA**	
**Gender**	**Mean ± SD**	**Mean ± SD**	***p***	${ƞ}_{p}^{2}$
**AOD500 (mm)**	0.92 ± 0.33	1.04 ± 0.41	0.030 *	0.032
**TIA500 (°)**	41.27 ± 7.97	42.58 ± 7.81	0.156	0.014
**IT750 (mm)**	0.54 ± 0.08	0.57 ± 0.10	0.201	0.011
**ARA500 (mm^2^)**	0.13 ± 0.05	0.16 ± 0.08	0.019 *	0.038
**TISA500 (mm^2^)**	0.15 ± 0.06	0.17 ± 0.08	0.062	0.024
**CCT (μm)**	543.20 ± 15.73	546.25 ± 27.33	0.515	0.003
**IOP (mmHg)**	15.68 ± 2.86	14.98 ± 1.95	0.349	0.006

* *p* < 0.05; ${ƞ}_{p}^{2}$: partial eta square effect value; SD: standard deviation; AOD: angle open distance; TIA: trabecular–iris angle; IT: iris thickness; ARA: angle recess area; CCT: central corneal thickness; IOP: intraocular pressure.

**Table 3:** Comparison of RNFL parameters in athletes and sedentary persons.

	Sedentary (n = 33)	Athletes (n = 42)	ANCOVA	
Group	Mean ± SD	Mean ± SD	*p*	${ƞ}_{p}^{2}$
**TOTAL (µm)**	107.87 ± 8.71	109.48 ± 8.63	0.671	0.001
**SUPERIOR (µm)**	135.23 ± 14.04	138.14 ± 10.27	0.277	0.008
**INFERIOR (µm)**	139.71 ± 15.15	140.05 ± 12.01	0.886	<0.001
**NASAL (µm)**	82.65 ± 12.84	86.70 ± 13.42	0.210	0.011
**TEMPORAL (µm)**	75.02 ± 7.61	76.21 ± 7.81	0.105	0.018
	**Right (n = 75)**	**Left (n = 75)**	**ANCOVA**	
**Side**	**Mean ± SD**	**Mean ± SD**	***p***	${ƞ}_{p}^{2}$
**TOTAL (µm)**	108.87 ± 8.88	108.29 ± 8.54	0.684	0.001
**SUPERIOR (µm)**	134.72 ± 12.85	138.29 ± 12.1	0.081	0.021
**INFERIOR (µm)**	140.27 ± 14.42	139.45 ± 13.26	0.721	0.001
**NASAL (µm)**	85.47 ± 12.1	83.40 ± 14.24	0.337	0.006
**TEMPORAL (µm)**	77.36 ± 7.79	73.73 ± 7.21	0.003 *	0.058
	**Female (n = 33)**	**Male (n= 42)**	**ANCOVA**	
**Gender**	**Mean ± SD**	**Mean ± SD**	***p***	${ƞ}_{p}^{2}$
**TOTAL (µm)**	108.65 ± 6.91	108.52 ± 9.9	0.662	0.001
**SUPERIOR (µm)**	136.5 ± 12.87	136.51 ± 12.4	0.666	0.001
**INFERIOR (µm)**	140.3 ± 11.59	139.51 ± 15.4	0.690	0.001
**NASAL (µm)**	82.50 ± 13.04	85.95 ± 13.22	0.277	0.008
**TEMPORAL (µm)**	76.44 ± 6.73	74.85 ± 8.35	0.097	0.019

* *p* < 0.05; ${ƞ}_{p}^{2}$: partial eta square effect value; SD: standard deviation.

## Data Availability

The datasets used and/or analyzed during the current study are available from the corresponding author upon reasonable request.
